# Uncoupled: investigating the lack of correlation between the transcription of putative plastic-degrading genes in the global ocean microbiome and marine plastic pollution

**DOI:** 10.1186/s40793-024-00575-4

**Published:** 2024-05-15

**Authors:** Victor Gambarini, Cornelis J. Drost, Joanne M. Kingsbury, Louise Weaver, Olga Pantos, Kim M. Handley, Gavin Lear

**Affiliations:** 1 School of Biological Sciences, University of Auckland, 3a Symonds Street, Auckland, 1010 New Zealand; 2 Centre for eResearch, University of Auckland, 23 Symonds Street, Auckland, 1010 New Zealand; 3 The Institute of Environmental Science and Research, 27 Creyke Road, Ilam, Christchurch, 8041 New Zealand

**Keywords:** Plastic pollution, Plastic biodegradation, Global ocean microbiome, Environmental ecology, Tara Oceans

## Abstract

**Background:**

Plastic pollution is a severe threat to marine ecosystems. While some microbial enzymes can degrade certain plastics, the ability of the global ocean microbiome to break down diverse environmental plastics remains limited. We employed metatranscriptomic data from an international ocean survey to explore global and regional patterns in microbial plastic degradation potential.

**Results:**

On a global oceanic scale, we found no significant correlation between levels of plastic pollution and the expression of genes encoding enzymes putatively identified as capable of plastic degradation. Even when looking at different regional scales, ocean depth layers, or plastic types, we found no strong or even moderate correlation between plastic pollution and relative abundances of transcripts for enzymes with presumed plastic biodegradation potential. Our data, however, indicate that microorganisms in the Southern Ocean show a higher potential for plastic degradation, making them more appealing candidates for bioprospecting novel plastic-degrading enzymes.

**Conclusion:**

Our research contributes to understanding the complex global relationship between plastic pollution and microbial plastic degradation potential. We reveal that the transcription of putative plastic-degrading genes in the global ocean microbiome does not correlate to marine plastic pollution, highlighting the ongoing danger that plastic poses to marine environments threatened by plastic pollution.

**Supplementary Information:**

The online version contains supplementary material available at 10.1186/s40793-024-00575-4.

## Background

Coastal and marine environments play a crucial role in our planet’s overall health and functioning [1]. These diverse ecosystems provide habitats for many species and provide essential services, such as carbon sequestration, sediment trapping and nutrient cycling. They also support various economic activities, including fisheries, tourism and recreation [2]. However, marine ecosystems face significant threats from human-induced pollution, with plastic pollution emerging as a severe and pressing concern [3, 4]. Plastic pollution directly impacts ecosystems by entangling marine mammals and birds and blocking their digestive tracts [5, 6], and indirectly by disrupting ecosystem structure and service provision [7]. Land-based sources account for 80% of plastics entering the oceans, with the majority coming from coastal mismanaged waste [8] but also via the overland flow of material, including particulate plastics resulting from the wear and tear of items such as tyres, road markings and the laundering of synthetic textiles [9]. Offshore activities, including commercial fishing, navigation, waste disposal and fish farming, introduce additional plastic debris directly into the marine environment, with damaged or discarded fishing gear posing a significant threat [10].

Plastic pollution exists in various forms. Primary microplastics are manufactured as small (< 5 mm diameter) particles. They are commonly found in cosmetics, medications, and air-blasting media. Secondary microplastics are formed through the breakdown of larger plastic debris [11] via the actions of ultraviolet radiation, physical abrasion and other environmental processes [12]. Thus, environmental plastics are frequently characterised by their size. ‘Megaplastic’ refers to plastic debris larger than 100 mm, and ‘macroplastic’ refers to debris ranging from 20 mm to 100 mm. Smaller plastics sizes include ‘mesoplastics’, which fall within the 5 mm to 20 mm range, ‘microplastics’ measuring between 1 mm and 5 mm, and ‘nanoplastics’ measuring less than 1 mm in size [13–15]. There are contrasting observations on the size distribution of these plastic particles in the ocean, with Cózar, et al. [16] suggesting our oceans are dominated by particles smaller than 10 mm, while Kaandorp, et al. [17] found that larger plastics (> 25 mm) contribute to more than 95% of the initially buoyant marine plastic mass. Although designed to degrade under controlled conditions, biodegradable plastics contribute to the microplastic fraction in aquatic environments [18].

Plastics may exhibit buoyancy, neutral buoyancy, or sinking behaviour depending on their composition and density, encouraging an initial vertical ‘sorting’ of different polymer types within the water column and on the seafloor [19, 20]. However, polymer density is by no means a dominant determinant of vertical plastic litter transport [21], as oceanographic factors, biofouling, and particle size impact the subsequent vertical transport of ageing plastics [22]. Choy, et al. [23] examined microplastic distribution in the Monterey Bay pelagic ecosystem and found that microplastics were dispersed throughout the water column, but peaked in abundance just below the mixed layer, at 250 m depth, and with the lowest concentrations observed at the ocean’s surface. In addition to vertical gradients of plastic pollution, plastic waste is not evenly distributed across the world’s major ocean basins. Instead, certain areas accumulate more plastic debris, such as within gyres, due to these circular currents trapping floating debris [24]. Indeed, gyres, including those within the North and South Pacific, North and South Atlantic, and the Indian Ocean, are well-known accumulation zones for floating plastic debris [25, 26].

Microbes are closely intertwined with the fate and impact of plastic pollution in the marine environment. The interaction between plastics and marine microorganisms has garnered increasing attention in recent years [27, 28]. Early reports of microbial colonisation on plastic surfaces date back to 1972 when Carpenter, et al. [29] documented such occurrences along the southern New England coast. Recent studies have increasingly focused on understanding the impacts of microplastics and plastic leachate on microbial community structure and functions [30]. Using omics, Zettler, et al. [31] revealed the widespread presence of microbial communities on plastic fragments dispersed throughout the North Atlantic Ocean, and coined the term “plastisphere” to describe these man-made ecological niches. While the durability of synthetic plastics allows them to persist in marine ecosystems, they can be slowly degraded by abiotic and biotic processes [12], where they can provide a carbon source for microbial growth. Consequently, microorganisms play a pivotal role in the degradation of certain plastics and their abiotic degradation products, across diverse marine habitats [32–34]. The microbial degradation of plastics in the marine environment is a complex and dynamic process. Microbes can metabolise and break down various plastic polymers [35, 36], potentially reducing the size and abundance of plastic particles. However, the extent and efficiency of microbial degradation of plastics in the ocean are still not fully understood [37]. Environmental conditions, plastic composition, and microbial communities’ diversity and activity can influence plastic degradation success and rates.

Recently, several large-scale research projects have collected extensive environmental and genetic data from marine ecosystems (e.g., Tara Oceans [38], International Census of Marine Microbes [39], and The Global Ocean Sampling Expedition [40]). The Tara Oceans Project stands out as one of the most influential efforts in this regard by collecting extensive environmental, metagenomic and metatranscriptomic data during multiple global voyages. These projects provide invaluable insights into the diversity and activities of marine microorganisms, shedding light on their complex community interactions [38]. However, the full potential of the data collected by these large-scale marine research projects remains largely untapped. Further exploration of the genomic capabilities for plastic degradation across global ecosystems is still needed to more fully utilise these rich datasets.

Zrimec, et al. [41] made the first contribution by finding a moderate correlation between the number of orthologous genes for plastic degradation and plastic pollution in the ocean. The results were based on the analysis of metagenome-assembled genomes (MAGs) from 41 samples collected by the Tara Oceans project. The genes in the MAGS were correlated to marine plastic pollution data published by four separate studies. However, ample opportunity remains to build on their work. The four projects quantified plastic pollution using distinct collection and measurement methodologies. The sampling sites also differed, with locations from Tara Oceans and the pollution studies separated by up to 400 km. The authors also excluded results from taxa related to the human gut microbiome from their analysis, although research indicates gut microbes, especially insect-associated ones, may degrade plastics. In fact, several microorganisms abundant in the human gut have demonstrated plastic-degrading abilities, such as *Clostridium* and Bacteroides [35], with recent research indicating plastic degradation occurs during simulated digestion within the human gut [42]. As pioneering work, Zrimec, et al. [41] paves the way for additional research to more fully elucidate genomic connections to marine plastic pollution across marine environments.

We sought to build upon the findings of Zrimec, et al. [41] by assessing relationships between modelled plastic concentrations in the global oceans and the transcription of potential plastic-degrading genes. Our analysis uses the recently released metatranscriptomic data collected by the Tara Oceans project, increasing the number of samples by an order of magnitude in comparison to Zrimec, et al. [41]. Additionally, we employ twice as many genes identified as having plastic-degrading activity, by using the expanded database provided by PlasticDB [35]. PlasticDB is a carefully curated database which compiles data on enzymes associated with plastic biodegradation. The data were obtained from a wide range of publications, providing information that includes details on microorganisms and proteins reported in the literature to degrade plastics and their metadata such as the sample sources and geographic location of microbial isolates. Finally, we do not insert any bias by excluding results from taxa also found within the human gut, as research suggests those taxa may also degrade plastics.

By leveraging the wealth of genetic data collected by the Tara Oceans project, and the collection of enzymes linked to plastic degradation within PlasticDB, we investigated correlations between the transcripts related to predicted plastic degradation traits and the concentrations and distributions of global marine plastic pollution. Through delving into this relationship, we sought to provide a deeper understanding of the microbial processes involved in plastic biodegradation and assess their potential as depolymerisation agents against the pervasive plastic pollution problem in our marine environments.

## Methods

### Metatranscriptomic data

Metatranscriptomic data were obtained from the Tara Oceans expedition [38], collected between 2009 and 2013. All the sample data are in the European Nucleotide Archive database, accessible at https://www.ebi.ac.uk/ena/browser/view/PRJEB402. Specifically, the accession numbers for the metatranscriptomic data are PRJEB6608 (465 samples) and PRJEB9741 (74 samples).

### Environmental data

Environmental data (e.g., water temperature and salinity, concentrations of oxygen, phosphate, nitrate, and nitrite in the water column.) were sourced from two primary locations: the supplementary information provided in the Tara Oceans publication [38] and additional data retrieved from the PANGEA database (https://doi.pangaea.de/10.1594/PANGAEA.875582).

### Data for enzymes putatively linked to plastic biodegradation

We used data on enzymes reported to degrade plastics that are collated within the PlasticDB database, accessible at https://plasticdb.org/downloaddata. This dataset comprises (on 01 May 2023) amino acid sequences for a total of 178 enzymes, covering 33 plastic types, including polyethylene terephthalate (PET), polycaprolactone (PCL), polyethylene (PE), polyurethane (PU), polylactic acid (PLA), and polyamide (PA, or ‘nylon’).

### Global marine plastic pollution data

We used a model of the global distribution of plastic pollution data published by Eriksen, et al. [43]. The authors estimated the total number of plastic particles and their weight at locations within the world’s oceans using data from 24 expeditions (2007–2013) across all five sub-tropical gyres, coastal Australia, the Bay of Bengal and the Mediterranean Sea. They conducted surface net tows (*N* = 680) and visual survey transects of large plastic debris (*N* = 891). The plastics were separated into four plastic size classes: 0.33–1.00 mm, 1.01–4.75 mm, 4.76–200 mm, and > 200 mm. The dataset, with plastic weight and count data for all collected samples, is available from 10.6084/m9.figshare.1015289, while the authors kindly shared the model itself upon contact.

### Bioinformatics analysis

The metatranscriptomic sequencing reads obtained by the Tara Oceans team were aligned against the PlasticDB protein reference database using the DIAMOND aligner [44]. The FASTA file downloaded from PlasticDB was indexed using the “diamond makedb” command with default parameters. The reads were aligned against the index using the “diamond blastx” command with the default parameters. The outputs from DIAMOND were parsed using Python and loaded into a pandas dataframe with each plastic as a column and each sample as a row. The cells contained the number of hits for each sample. Each value was normalised by the size of the library using the equation below.

$$normalised\,value = \frac{{number\,of\,hits}}{{\frac{{library\,size}}{{{\rm{1}}{\rm{.000}}{\rm{.000}}{\rm{.000}}}}}}$$

### Statistical analysis

All statistical analyses were performed in Python version 3.10.12 [45]. A one-way Analysis of Variance (ANOVA) was performed to assess overall differences among groups, testing the variables oceanic regions (North Atlantic Ocean, South Pacific Ocean, North Pacific Ocean, Indian Ocean, South Atlantic Ocean, Arctic Ocean, Red Sea, Mediterranean Sea, Southern Ocean) and depth layers (surface water layer (SRF), deep chlorophyll maximum (DCM), mesopelagic zone (MES), mixed layer (MIX), marine water layer (ZZZ, this artificial layer contains samples that were not classified as belonging to the other layers)). Subsequently, Tukey’s Honestly Significant Difference (HSD) test was applied as a post hoc analysis to identify specific groups that exhibited statistically significant mean differences. The ANOVA and Tukey’s HSD test were conducted using Python with the scipy.stats (version 1.11.3; Virtanen, et al. [46]) and statsmodels (version 0.14.0; Seabold and Perktold [47]) libraries, respectively. The chosen significance level was α = 0.05 for both tests.

Correlation analysis was performed to assess the strength and direction of the relationship between pairs of variables (i.e., to compare relationships between the plastic and transcript data). This analysis was carried out using the pearsonr function from the scipy.stats (version 1.11.3; Virtanen, et al. [46]) module in Python. The pearsonr function calculates the Pearson correlation coefficient I and the associated two-tailed *p*-value. The resulting correlation coefficient, r, was interpreted as follows: values closer to 1 or -1 indicate a stronger correlation, while values near 0 suggest a weaker or no linear correlation. The associated *p*-value was used to assess the statistical significance of the observed correlation.

## Results

**Fig. 1:**
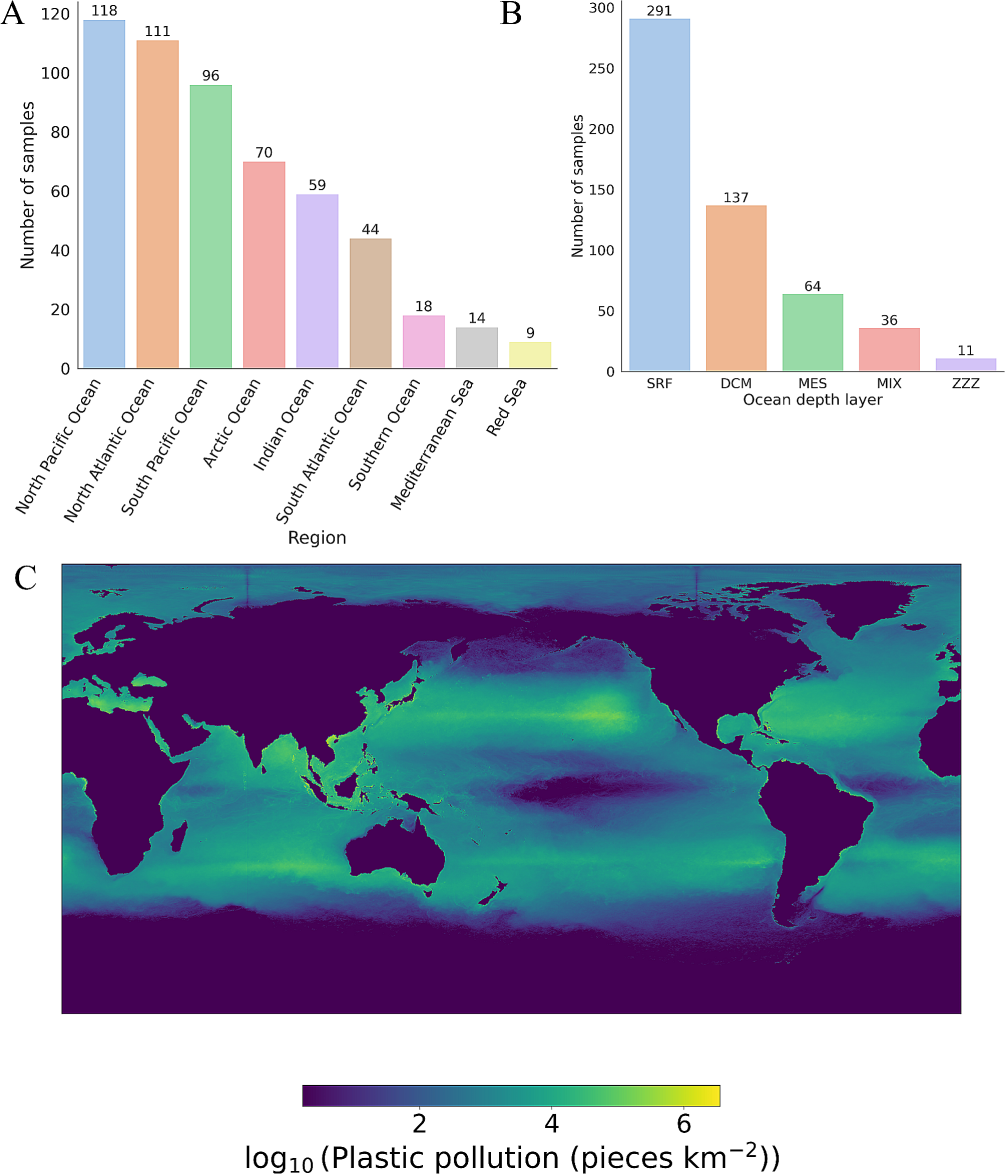
Samples collected by the Tara Oceans project: (**A**) number of samples by region, (**B**) number of samples by depth layer. (**C**) Distribution of marine plastic pollution according to a model published by Eriksen, et al. [43]

We obtained 539 metatranscriptomic samples from the Tara Oceans project [38]. Their sampling of the ocean microbiome was conducted across nine oceanic regions (Fig. 1A) and five depth layers, which included (i) the surface water layer (SRF; mean standard deviation [SD] of 3 ± 0 m, 291 samples), (ii) the deep chlorophyll maximum (DCM; 68 ± 42 m, 137 samples) layer, (iii) the mesopelagic zone (MES; 501 ± 173 m, 64 samples), (iv) a mixed layer (MIX; 42 ± 44 m, 36 samples), and (v) marine water layer (ZZZ; 86 ± 58 m, 11 samples) (Fig. 1B). All Tara Oceans RNA samples available were sequenced from size fractions ranging from 0.22 to 1.6 μm or 0.22–3.0 μm. The North Pacific Ocean had the most samples collected (118 samples), while at the depth level, the majority of the samples were collected from the surface layer.

To annotate the potential for plastic biodegradation in the metatranscriptome samples, we employed PlasticDB’s protein reference. Most of the enzymes contained in PlasticDB were reported to have activity against polyhydroxyalkanoate (PHA), polyhydroxybutyrate (PHB) polyethylene terephthalate (PET), polycaprolactone (PCL), and polylactic acid (PLA) plastics. To correlate transcripts for plastic degradation with actual plastic pollution, we obtained the model of plastic pollution in marine environments published by Eriksen, et al. [43] to assess the overall count and weight of plastic particles suspended in the global oceans (Fig. 1C). Marine plastic pollution appeared higher in two bands roughly aligned with the Tropics of Cancer and Capricorn, while the waters around Antarctica appeared to have the lowest levels of plastic pollution.

**Fig. 2:**
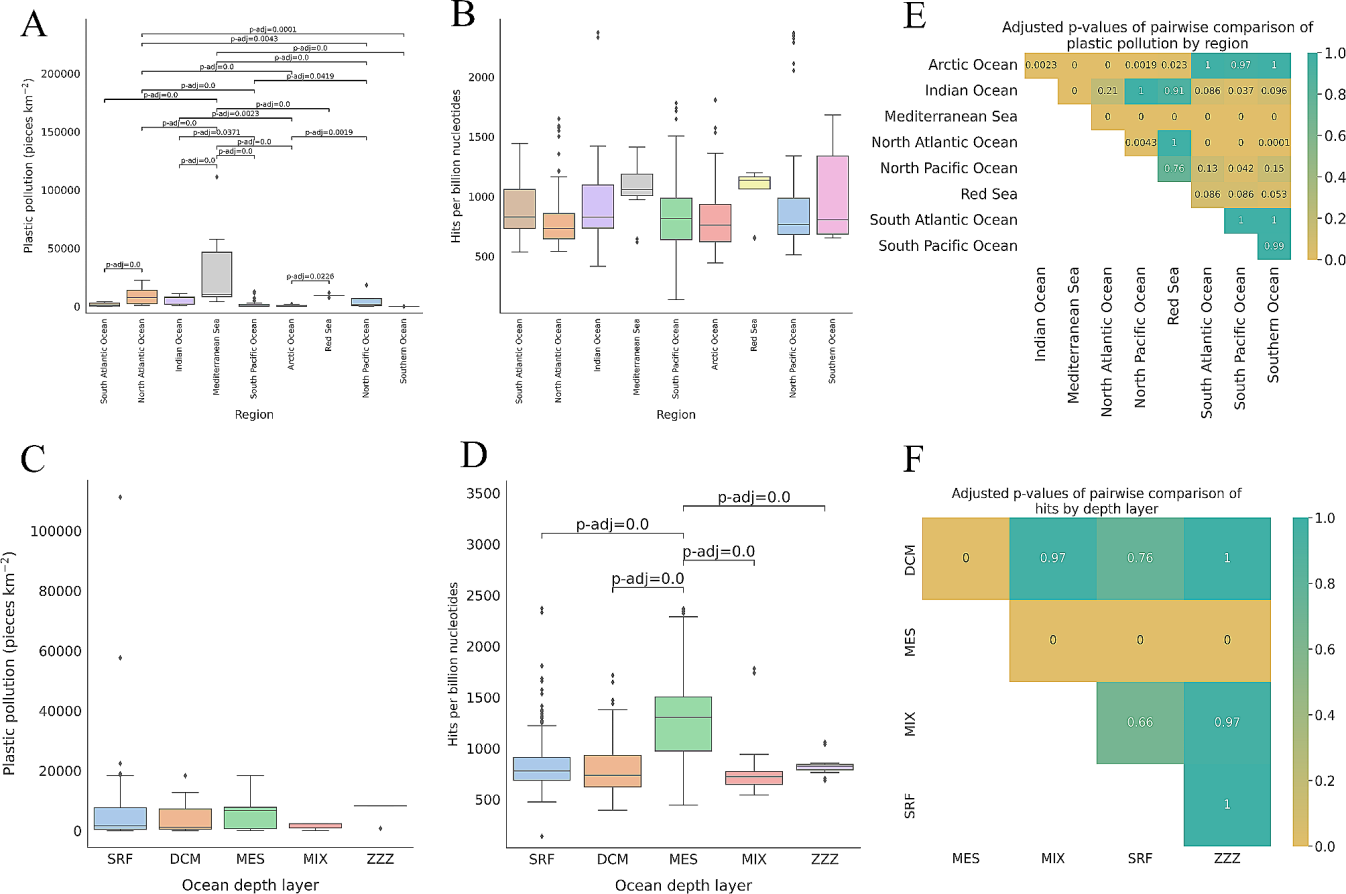
Summary of plastic pollution and relative abundances of enzymes linked to plastic degradation among different ocean regions and depth layers within the points where Tara Oceans samples were collected. **A** and **C**) plastic pollution split into oceanic regions and depth layers, respectively. Plastic pollution values are pieces km^− 2^. **B** and **D**) Normalised ‘hits’ for enzymes linked to plastic degradation, according to the procedure described in the methods, split into oceanic regions and ocean depth layers, respectively. Statistical analysis (ANOVA) reveals significant differences, overall, for all groups shown in boxplots (*p* < 0.05). Adjusted *p*-values on pairwise boxplot comparisons were obtained by applying Tukey’s Honestly Significant Difference test. Significant pairwise comparisons are shown on the boxplot where found. **E**) *P*-values of pairwise comparisons among samples split by region, comparing plastic pollution count means. F) *P*-values of pairwise comparisons among samples split by depth layers, comparing enzyme hit means

Using the plastic pollution model, we assigned a value of plastic pollution to each metatranscriptomic sample based on its geographic location. Within our samples, the Mediterranean Sea was the oceanic region where samples had the most plastic pollution (30,602 pieces km^− 2^). The least amount of plastic was found at sample locations in the Southern Ocean (1.76 pieces km^− 2^) (Fig. 2A). There were significant differences in plastic pollution among oceanic regions (ANOVA *p*-value: 4.26e-41); significant differences occurred for 18 out of 36 pairwise comparisons (Tukey’s HSD test; *p*-value < 0.05). On the other hand, when comparing different water layers, there were no significant differences (Tukey’s HSD test; *p*-value < 0.05) (Fig. 2C).

**Fig. 3:**
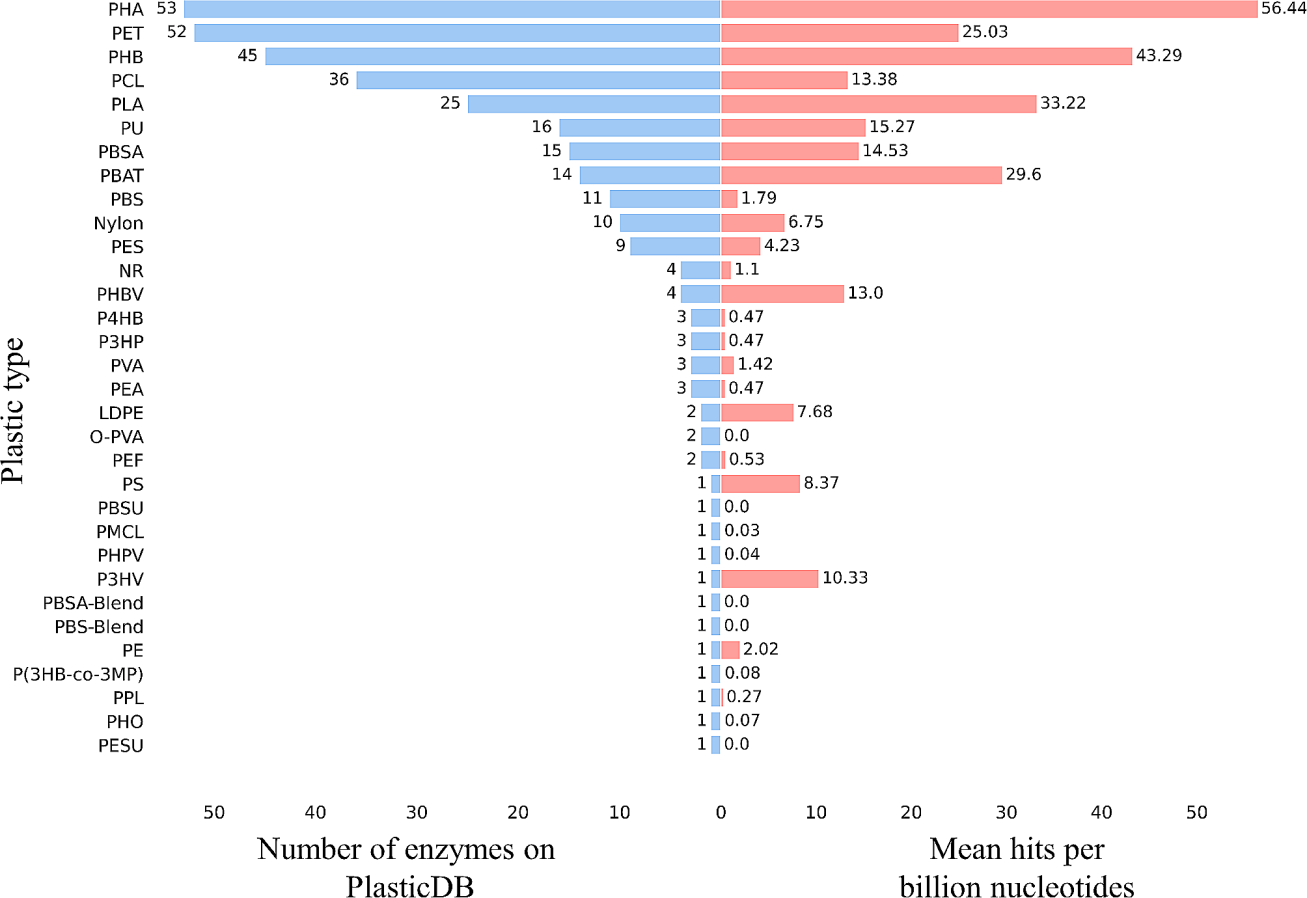
Enzymes downloaded from PlasticDB with reported activity against 32 different plastic types versus the mean number of hits per billion nucleotides for each plastic type. Abbreviations for all plastic names are in Supplementary Table 1

We annotated all metatranscriptomic data for their plastic biodegradation potential by using the data available within the PlasticDB protein reference database. Plastic types with higher representation in the PlasticDB dataset were found more frequently in the metatranscriptomic samples (Fig. 3). The oceanic region with the greatest number of enzyme hits was the Mediterranean Sea (1058 hits per billion nucleotides), while the least number of enzyme hits was found in the North Atlantic Ocean (799 hits per billion nucleotides) (Fig. 2B). The differences in the number of enzyme hits among regions were not as dramatic as the plastic pollution values, which differed by four orders of magnitude. In addition, the least plastic-polluted oceanic area, the Southern Ocean, had the third greatest number of hits for putative plastic-degrading enzymes (1000 hits per billion nucleotides).

**Fig. 4:**
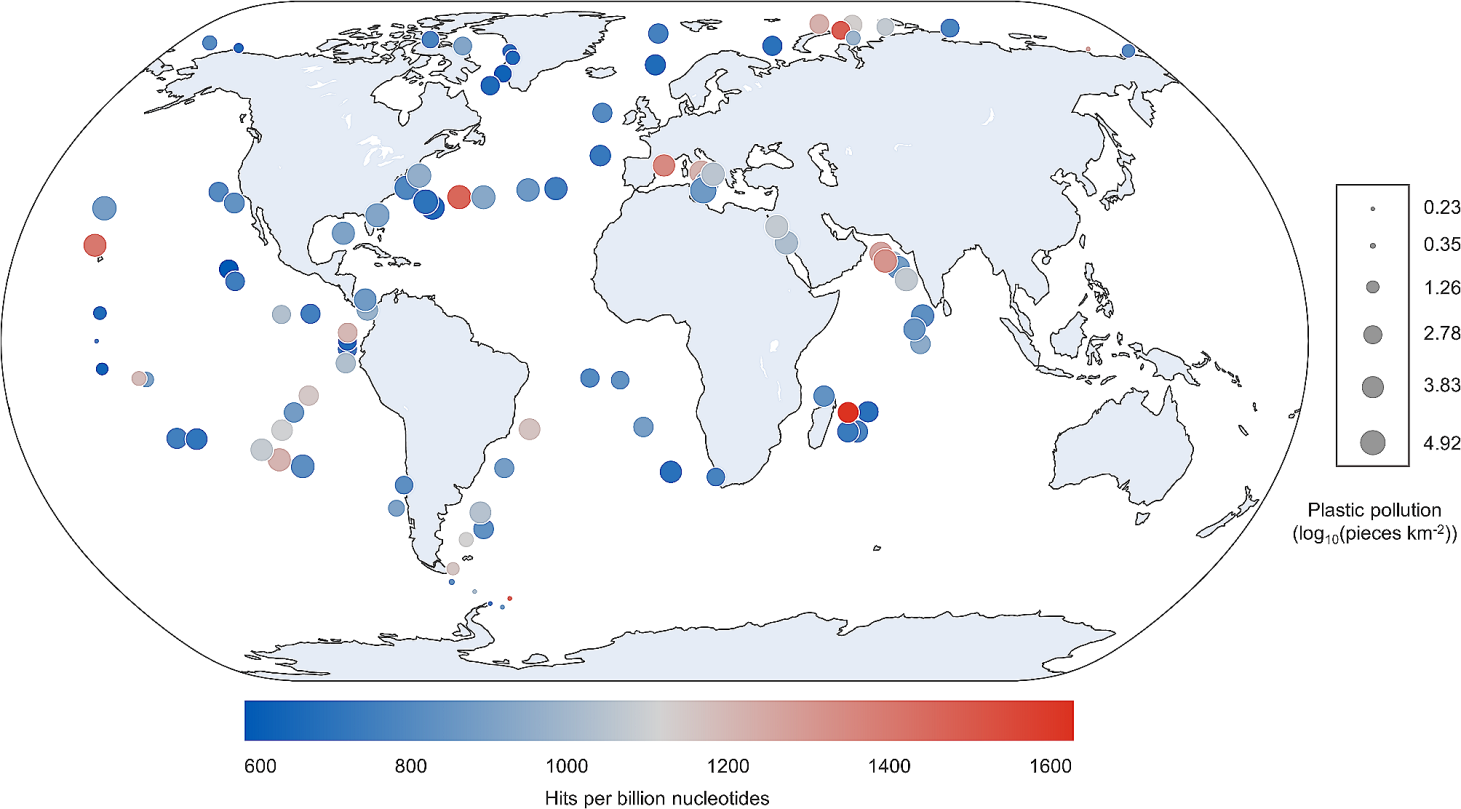
Global distribution of plastic pollution and hits for putative plastic degrading enzymes on samples collected by the Tara Oceans project. The size of the circle represents the amount of plastic pollution (log_10_ transformed), and the colour represents the number of hits. For visualisation purposes, samples within a 200 km radius were merged, and the enzyme hits and plastic pollution values were averaged

Using all 539 samples from nine oceanic regions and five different depths, a correlation was not found between ocean pollution and the expression of genes linked to plastic biodegradation (*r* = 0.05; *p*-value = 0.24; Fig. 4). For example, samples with small amounts of plastic and high numbers of enzyme hits were identified near the Southern Ocean, while samples with high plastic concentrations, but small numbers of enzyme hits were observed in the North Atlantic Ocean.

**Fig. 5:**
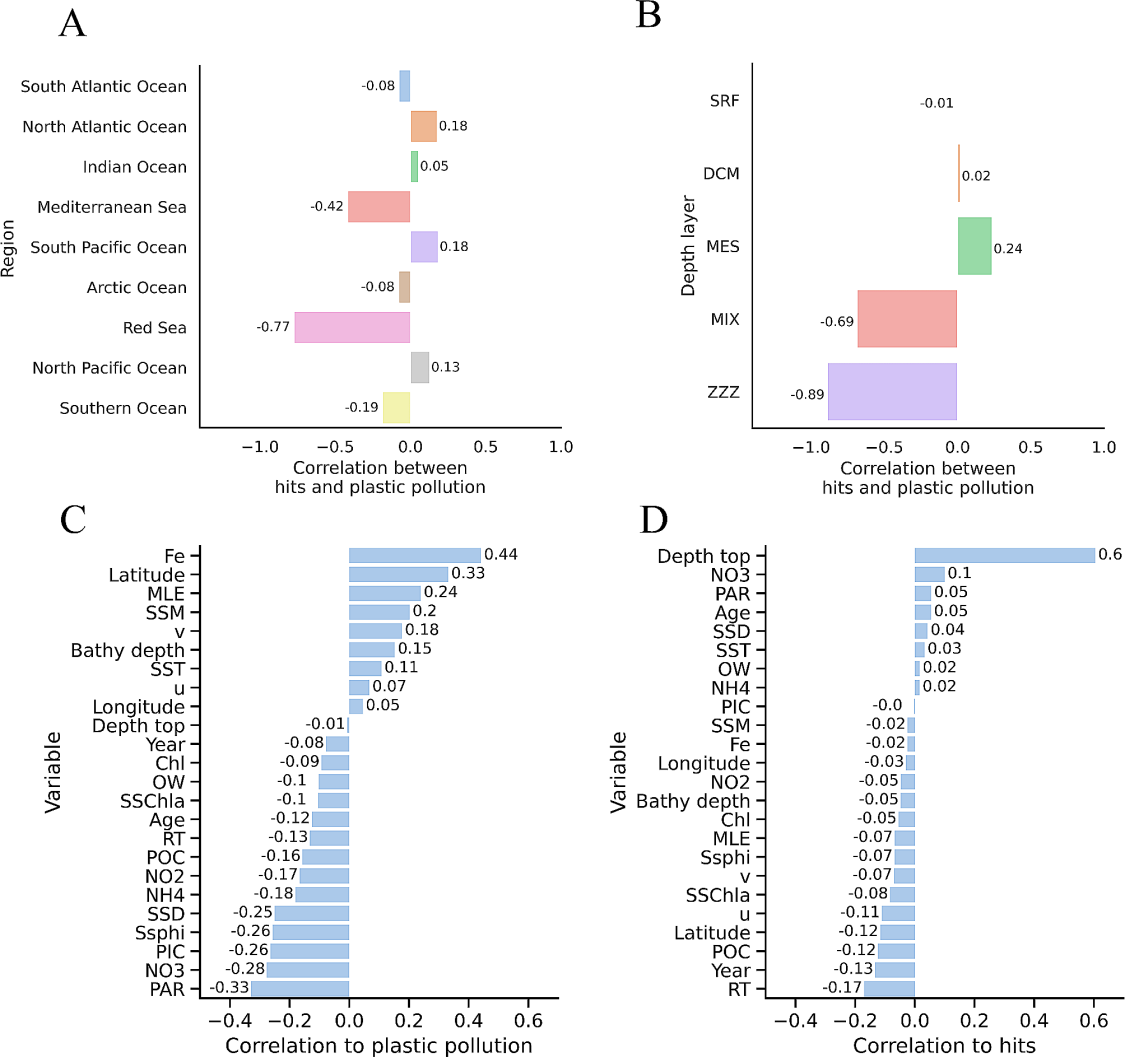
A and **B**) Pearson correlation between hits for plastic-degrading enzymes and amounts of plastic pollution in marine ecosystems, analysed by oceanic region or depth layers. **C** and **D**) Pearson correlation of plastic pollution and hits to environmental variables. Abbreviations: SRF (surface water layer), DCM (deep chlorophyll maximum), MES (mesopelagic zone), MIX (mixed layer), ZZZ (marine water layer), Fe (Iron), MLE (Maximum Lyapunov exponent), NO3 (Nitrate), and PAR (Radiation, photosynthetically active per day). Other abbreviations for environmental variables are in Supplementary Table 2

To investigate if there were significant correlations between plastic pollution in the oceans and the expression of genes linked to plastic biodegradation within subgroups of the larger dataset, we analysed the data according to oceanic regions or depth layers. When looking at oceanic regions, we observed a weak positive correlation in the South Pacific Ocean (*r* = 0.18; *p*-value = 0.07; 96 samples), a weak positive correlation in the North Atlantic Ocean (*r* = 0.18; *p*-value = 0.06; 111 samples) and North Pacific Ocean (*r* = 0.12; *p*-value = 0.17; 118 samples) (Fig. 5A). Conversely, we found a strong negative correlation in the Red Sea (*r*=-0.77; *p*-value = 0.01; 9 samples). When looking at the depth layer categories, the only positive correlations were the weak correlations for the mesopelagic zone (MES) layer (*r* = 0.24; *p*-value = 0.06; 64 samples) the deep chlorophyll maximum (DCM) layer (*r* = 0.02; *p*-value = 0.8; 137 samples) (Fig. 5B). We also found a very strong negative correlation in the marine water layer (ZZZ) (*r*=-0.89; *p*-value = 2.48e-04; 11 samples) and a strong negative correlation in the mixed layer (MIX) (*r*=-0.69; *p*-value = 4.0e-06; 36 samples).

As the Tara Oceans project samples are associated with various environmental measurements, we investigated the correlation between these ecological variables and plastic pollution or hits for plastic-degrading enzymes. When looking at positive correlations to plastic pollution (Fig. 5C), iron (Fe) moderately correlates to plastic pollution (*r* = 0.44; *p*-value = 1.66e-15), while latitude weakly correlates (*r* = 0.33; *p*-value = 5.29e-9). On the other hand, photosynthetically active radiation (PAR) (*r*=-0.33; *p*-value = 6.85e-9) and nitrate (NO_3_) (*r*=-0.28; *p*-value = 1.42e-6) had a weak negative correlation to plastic pollution.

We also analysed correlations between environmental variables and hits for plastic-degrading enzymes (Fig. 5D). The depth from the water’s surface (Depth top) moderately correlated to enzyme hits (*r* = 0.60; *p*-value = 7.52e-31). Conversely, the two greatest negative correlations to enzyme hits were very weak correlations: sampling year (Year) (*r*=-0.13; *p*-value = 2.21e-02) and age of water mass in days, measured as residence time (RT) (*r*=-0.16; *p*-value = 3.41e-03).

**Fig. 6:**
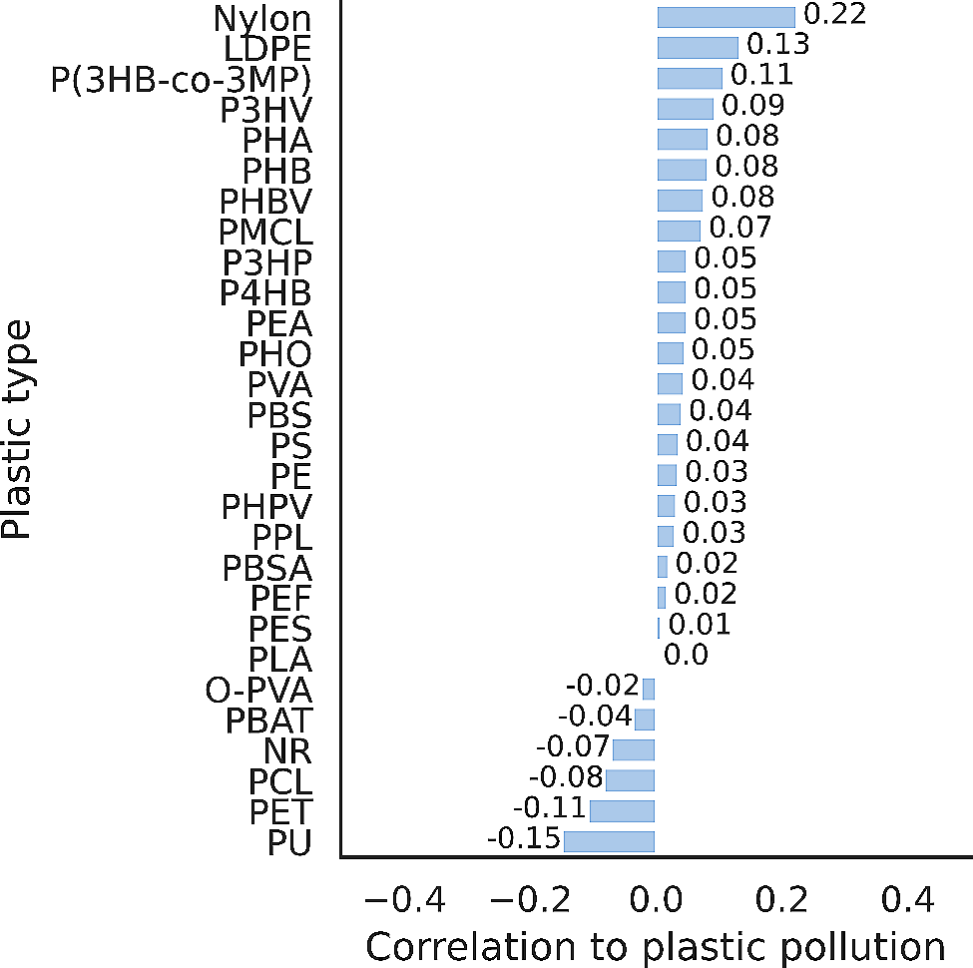
Pearson correlation between hits for putative plastic-degrading enzymes and amounts of plastic pollution in marine ecosystems, analysed by plastic type. Putative plastic-degrading enzymes were annotated according to PlasticDB. All samples from all oceanic regions and all depth layers were used to perform this analysis (*n* = 539). Abbreviations: LDPE (low density polyethylene), P(3HB-co-3MP) (Poly(3-hydroxybutyrate-co-3-mercaptopropionate)), P3HV

To explore potential connections between enzymes for degradation of specific plastic types and marine plastic pollution, we extracted the number of hits for each plastic type from our dataset (Fig. 6). The most significant positive correlations were found with nylon (*r* = 0.22; *p*-value = 1.68e-07) and LDPE (*r* = 0.13; *p*-value = 2.03e-03). The most substantial negative correlations were observed with PU (*r*=-0.15; *p*-value = 5.21e-04) and PET (*r*=-0.11; *p*-value = 1.19e-02).

(Polyhydroxyvalerate), and PHA (Polyhydroxyalkanoate). Other abbreviations for environmental variables are in Supplementary Table 1.

## Discussion

We sought to explore the relationship between plastic pollution in marine environments and the expression of genes associated with plastic degradation using metatranscriptomic data from the Tara Oceans project [38], marine plastic pollution data from Eriksen, et al. [43], and a database of enzymes linked to plastic biodegradation from the PlasticDB database [35]. Despite the abundance of plastic pollution in various oceanic regions and depth layers, this study did not find a significant global correlation between the expression of genes reported to encode plastic-degrading enzymes and plastic pollution levels. Our results indicate that on the global level, plastic pollution in the oceans and the expression of genes linked to plastic biodegradation are unrelated phenomena (*r* = 0.05; *p*-value = 0.24).

These results likely indicate that the global ocean microbiome has not yet developed widespread abilities to biodegrade synthetic polymers. These results are in accordance with the current literature on microbial plastic degradation, as synthetic polymers with a pure carbon backbone appear not to be degraded by microbes, even under extremely favourable laboratory conditions [48]. These plastics, including polypropylene (PP), low-density polyethylene (LDPE), high-density polyethylene (HDPE), polyvinyl chloride (PVC) and polystyrene (PS), also called homochain polymers, are mainly produced from petroleum-based materials and are by far the most produced and discarded types of plastics worldwide [49]. Plastics containing atoms other than carbon in the main chain are called heterochain polymers; these include polymers such as nylon, poly(ethylene terephthalate) (PET), and polyurethane (PU). These plastics are more biodegradable than homochain polymers; however, the literature shows in some cases that only amorphous regions of these plastics are degraded by microorganisms, and the highly crystalline areas of the plastic are degraded little or not at all [50]. Plastic biodegradation may also be influenced by the presence of other carbon sources more easily utilized by microbial cells. Such phenomenon has been largely reported in the literature [51–53].

Further analysis at regional and depth-specific levels uncovered specific correlations between plastic pollution and the expression of plastic biodegradation genes. For instance, both the South (*r* = 0.18; *p*-value = 0.07; 96 samples) and the North Pacific Ocean (*r* = 0.13; *p*-value = 0.16; 118 samples) exhibited a weak positive correlation, but the correlations were not statistically significant at a significance level of 0.05. The only statistically significant correlation was the strong negative correlation found in the Red Sea (*r*=-0.77; *p*-value = 0.01; 9 samples). It should be noted that transforming the plastic pollution data, for example through logarithmic approaches, did not substantially alter the correlations and *p*-values reported here, showing our results to be rigorous regardless of data transformation approaches. The Red Sea exhibits uniquely high salinity and temperature compared to other oceanic regions [54]. These or other geographical and physical attributes may select for Red Sea microbial populations with less genetic plastic degradation potential. However, the number of samples collected in the Red Sea was very limited and further investigation into regional drivers of microbial genomic adaptations regarding plastic metabolism are needed to help elucidate the relationships suggested by these findings.

We found a weak negative correlation between the relative abundance of enzymes encoded that are presumed capable of plastic degradation and plastic pollution in the Southern Ocean (*r*=-0.19; *p*-value = 0.46; 18 samples), although the *p*-value was not significant, likely due to the low number of samples obtained for this region. Interestingly, despite being the least polluted ocean (1.76 pieces km^− 2^), the Southern Ocean had the third highest number of hits for potential plastic-degrading enzymes (1000 hits per billion nucleotides). Therefore, the Antarctic region stands out as a promising location for the potential discovery of new plastic-degrading enzymes. This observation suggests that areas with the least plastic waste may also be good candidates for bioprospecting novel plastic-degrading enzymes, challenging the current trend that typically directs sampling efforts towards highly polluted areas.

Inverse relationships between plastic pollution and relative abundances of genes putatively encoding for plastic degradation were also observed in our study. Notably, the Red Sea exhibited a strong negative correlation (*r*=-0.77; *p*-value = 0.01; 9 samples), while the Mediterranean Sea displayed a moderate negative correlation (*r*=-0.42; *p*-value = 0.13; 14 samples). However, it is important to note that the low number of samples collected in both locations likely contributed to the high *p*-value results. Specific environmental conditions, ecological factors, or limitations in the dataset may influence this unexpected result. It emphasises the complexity of the relationship between plastic pollution and microbial plastic degradation, which can vary across different geographic locations and environmental contexts.

The depth-specific analysis provided additional insights into the dynamics of plastic pollution and microbial responses. The mesopelagic zone (MES) had a moderate positive correlation between plastic pollution and hits for putative plastic-degrading enzymes (*r* = 0.24; *p*-value = 0.06; 64 samples). This oceanic layer was found by Choy et al. (2019) to have the highest amounts of microplastic in the ocean water column. These results imply that microbial communities at this depth might play a more active role in plastic degradation than other layers in response to their higher exposure to microplastics. On the contrary, the samples collected from a depth labelled as marine water layer (ZZZ; 86 ± 58 m, 11 samples that were not classified in any of the other layers) exhibited a very strong negative correlation (*r*=-0.89; *p*-value = 2.48e-04), indicating a unique environment or microbial community structure in this ‘layer’ that prevent a greater abundance of genes encoding putative plastic-degrading enzymes. These depth-specific findings underscore the importance of considering vertical dimensions in understanding the intricate relationships between microbial communities and ocean plastic pollution.

The environmental variable analysis provided additional insights into factors influencing plastic pollution and microbial plastic degradation. Iron (Fe) and latitude positively correlated with plastic pollution. This phenomenon may be explained by the ocean distribution of iron and plastic pollution both being influenced by the same factors, most likely aeolian processes that transport particles long distances [55, 56]. This correlation between iron and plastic pollution may become a serious environmental issue. Iron availability is a key nutrient for cyanobacteria growth [57], and it has been shown that microplastics may be toxic to the most abundant photosynthetic organism on Earth, the marine cyanobacteria *Prochlorococcus* [58]. Photosynthetically active radiation (PAR) and nitrate (NO_3_^−^) displayed a weak negative correlation with plastic pollution, highlighting the potential association, causative or not, of nitrogen concentrations with plastic accumulation. In terms of plastic degradation-related hits, a moderate positive correlation was observed with the depth from the top environmental variable. Microbial communities are known to demonstrate vertical stratification and differentiation with depth in marine environments due to shifts in light, nutrients, pressure and other factors [38]. Surface communities harbour more phototrophs while deep communities include more chemoautotrophs and heterotrophs [59]. The increase in plastic-degradation hits with depth could reflect an enrichment of microbial taxa in deeper strata that contain a higher repertoire of hydrolytic, heterotrophic and other enzymes capable of breaking down plastics and other organic matter.

Although the PlasticDB dataset tries to compile all known information on enzymes with plastic degradation activity, some plastic types are more represented than others. To account for this proportionally different representation of different plastic types, our analysis also considered the correlation of plastic pollution with the expression of genes encoding enzymes with the potential for the degradation of different plastic types. We did not find any moderate or strong correlation for any of the plastic types present in our dataset. For example, nylon (*r* = 0.22; *p*-value = 1.68e-07) and LDPE (*r* = 0.13; *p*-value = 2.03e-03) both had weak positive correlations. Conversely, we found weak negative correlations for PU (*r*=-0.15; *p*-value = 5.21e-04) and PET (*r*=-0.11; *p*-value = 1.19e-02). PlasticDB primarily covers well-characterized plastic-degrading enzymes and, therefore, may not take into account the potential role of promiscuous enzymes as discussed in Zadjelovic, et al. [60]. These enzymes, which may generate reactive oxygen species or catalyse non-specific oxidation reactions, could contribute to the initial depolymerisation of recalcitrant plastics, facilitating subsequent assimilation by microorganisms.

All Tara Oceans RNA samples available were sequenced from size fractions ranging from 0.22 to 1.6 μm or 0.22–3.0 μm. Therefore, as plastics occur in the ocean in a range of sizes, from meters to nanometres, the relative abundance of these plastic types in the sequenced size fractions likely reflects their general relative abundance in the ocean. Overall, these findings indicate the global ocean microbiome has not yet adapted to biodegrade any specific types of plastic. Microbial adaptation to plastics may also be constrained by the relatively low energy yield upon enzymatic degradation, limiting the selective advantage for microbes to degrade it. The lack of a significant global correlation highlights the need for a nuanced understanding of the factors influencing microbial plastic degradation. Plastic pollution is a multifaceted issue influenced by various factors, including polymer type, environmental conditions, and possibly by the diversity of microbial communities. Microbial adaptation to plastic pollution may be a localised phenomenon, and regional differences in plastic types, microbial communities, and environmental variables can contribute to the observed variations.

In conclusion, this research contributes to understanding the intricate relationship between plastic pollution and microbial plastic degradation globally, indicating that the global ocean microbiome has not yet adapted to biodegrade plastic pollution. The findings also highlight the importance of considering regional variations, and the urgent need to increase sampling and research efforts in understudied oceanic regions. Our data suggest that microorganisms in Antarctic environments may have high potential for plastic degradation, making them promising candidates for future research on new plastic-degrading enzymes. By shedding light on these nuanced interactions, our study adds a crucial layer to the ongoing discourse on plastic pollution, providing a foundation for developing strategic interventions to mitigate the environmental impact of plastic waste in our oceans.

### Electronic supplementary material

Below is the link to the electronic supplementary material.

Supplementary Material 1

## Data Availability

All data and analysis code are available in the GitHub repository here: https://github.com/VictorGambarini/GlobalPlastic.
