# Supplementary material for: Uncoupled: investigating the lack of correlation between the transcription of putative plastic-degrading genes in the global ocean microbiome and marine plastic pollution
Source: Environ Microbiome. 2024 May 15;19:34. doi: 10.1186/s40793-024-00575-4 (PMC11097532; doi:10.1186/s40793-024-00575-4)
Supplement: Supplementary file 1 — Supplementary Material 1 [file 40793_2024_575_MOESM1_ESM.docx]

**Supplementary Table 1**: Plastic abbreviations

| **Abbreviation** | **Full polymer name** |
| --- | --- |
| PE | Polyethylene |
| PET | Polyethylene terephthalate |
| PS | Polystyrene |
| PP | Polypropylene |
| PLA | Polylactic acid |
| PU | Polyurethane |
| PVC | Polyvinyl chloride |
| PHA | Polyhydroxyalkanoate |
| PHB | Polyhydroxybutyrate |
| PHBV | Poly(3-hydroxybutyrate-co-3-hydroxyvalerate) |
| P4HB | Poly(4-Hydroxybutyrate) |
| P3HP | Poly(3- hydroxypropionate) |
| P3HV | Polyhydroxyvalerate |
| PCL | Polycaprolactone |
| PBAT | Polybutylene adipate terephthalate |
| PBS | Polybutylene succinate |
| PBSA | Polybutylene succinate adipate |
| PES | Polyethylene succinate |
| PEG | Polyethylene glycol |
| NR | Natural rubber |
| PEA | Polyethylene adipate |
| PVA | Polyvinyl alcohol |
| P(3HB-co-3MP) | Poly(3-hydroxybutyrate-co-3-mercaptopropionate) |
| PEF | Polyethylene-2,5- furandicarboxylate |
| PPL | Polypropiolactone |
| PMCL | Poly(γ-methyl-ε-caprolactone) |
| PHO | Poly(3-hydroxyoctanoate) |
| PHPV | Polyhydroxyphenylvalerate |
| PETG | Polyethylene Terephthalate Glycol |
| P34HB | Poly(3-hydroxybutyrate-co-4-hydroxybutyrate) |
| PBST55 | Poly(butylene succinate-co-terephthalate)55 |
| PBSTIL | Poly(butylene succinate-terephthalate-isophthalate-co-lactate) |
| PC | Polycarbonate |
| PSS | Polystyrene sulfonate |
| PTC | Poly(tetramethylene carbonate) |
| PHC | Poly(hexamethylene carbonate) |
| PTS | Poly(tetramethylene succinate) |

**Supplementary Table 2**: Environmental variables abbreviations

| **Abbreviation** | **Full variable name** |
| --- | --- |
| Fe | Iron, total |
| MLE | Maximum Lyapunov exponent |
| SSM | Strain sub-mesoscale index |
| v | Geostrophic velocity, latitudinal |
| SST | Sea surface temperature |
| u | Geostrophic velocity, longitudinal |
| Chl | Chlorophyll a |
| OW | Okubo-Weiss paramete |
| SSChla | Sea surface chlorophyll a |
| RT | Residence time |
| POC | Particulate organic carbon |
| NO2 | Nitrite |
| NH4 | Ammonium |
| SSD | Sunshine duration |
| Ssphi | Sea surface quantum yield of fluorescence |
| PIC | Carbon, inorganic, particulate |
| NO3 | Nitrate |
| PAR | Radiation, photosynthetically active per day |
